# Regional differences in performance of bone marrow transplantation, care-resource use and outcome for adult T-cell leukaemia in Japan

**DOI:** 10.1186/1472-6963-14-337

**Published:** 2014-08-08

**Authors:** Toshiki Maeda, Akira Babazono, Takumi Nishi, Shinya Matsuda, Kiyohide Fushimi, Kenji Fujimori

**Affiliations:** 1 Department of Healthcare Administration and Management, Graduate School of Healthcare Sciences, Kyushu University, 3-1-1 Maidashi, Higashi-ku, Fukuoka 812-8582, Japan; 2 Department of Preventive Medicine and Community Health, University of Occupational and Environmental Health, 1-1, Iseigaoka, Yahata-nishi-ku, Kitakyushu, Fukuoka 807-8555, Japan; 3 Department of Health Policy and Informatics, Tokyo Medical and Dental University, Graduate School of Medicine, 1-5-45, Yushima, Bunkyo-ku, Tokyo 113-0034, Japan; 4 Department of Health Administration and Policy, Tohoku University, 2-1 Seiryo-machi, Aoba-ku, Sendai, Miyagi 980-8575, Japan

**Keywords:** Adult T-cell leukaemia, Regional differences, Bone marrow transplantation, Care-resource, Outcome, Hospital administrative database

## Abstract

**Background:**

Japan has a high prevalence of adult T-cell leukaemia (ATL), especially in the Kyushu/Okinawa region. Regional differences in prevalence might cause regional differences in physicians’ experiences and the efficiency of care-resource use. This study investigated regional differences in the performance of bone marrow transplantation (BMT), outcome and care-resource use in patients with ATL in Japan.

**Methods:**

This was a cross-sectional study using a Japanese hospital administrative database in 2010, with a diagnostic-procedure combination/per diem payment system. We examined the association between BMT performance, resource use, outcomes and region.

**Results:**

We analysed data for 712 subjects of whom 60.5% were Kyushu/Okinawa residents. Significantly more patients with ATL underwent BMT in Kanto (p = 0.018) and Kansai (p < 0.001) regions compared with the Kyushu/Okinawa regions. The lengths of hospital stay were longer in Kanto (p = 0.002) and Kansai (p = 0.006) regions than in the Kyushu/Okinawa region. Total health-care costs were higher in Kanto (p = 0.001) and Kansai (p = 0.005) regions than the Kyushu/Okinawa region. The risks of in hospital mortality were not significantly different between regions.

**Conclusions:**

There were significant regional differences in BMT performance and resource use within Japan. ATL prevalence was not related to the performance of BMTs, resource use or outcomes. Factors related to regional socioeconomics might affect the performance of BMTs and care resource use within Japan.

## Background

Adult T-cell leukaemia (ATL) was initially described by Takatsuki et al. in 1977 [1]. ATL is characterized by an increase in mature T cells following the insertion of human T-lymphotropic virus 1 (HTLV-1) into chromosomal DNA, and only occurs in HTLV-1 carriers.

HTLV-1 infection is transmitted to newborns by breast milk from HTLV-1 carrier mothers [2]. ATL occurs in less than 5% of people with HTLV-I infection, with a mean latency period of more than 30 years [3]. ATL is classified into four categories [4]. The median survival time ranges from 3.7 to 6.0 months for the acute and lymphomatous forms, while the median survival is 2 years or more in indolent smouldering and chronic forms [3]. The median survival time of those treated by chemotherapy was 13 months [5], thus it is unfavourable compared to other hematologic malignancies. Accordingly, bone marrow transplantation (BMT) is a promising therapy associated with long-term survival [5].

The prevalence of ATL in Japan is one of the highest worldwide [6]. A national survey conducted in the 1980s reported that HTLV-1 carriers were concentrated in the southwest of Japan, with approximately 700 newly diagnosed ATL patients per year [7]. Although patients with ATL have a poor prognosis, the government concluded that the prevalence of ATL would decrease because of low infectivity and limited routes of infection, and therefore, national investigations were discontinued [8]. However, the Ministry of Health, Labour and Welfare (MHLW) conducted a national survey of HTLV-1 antibodies in newly donated blood samples from 2007 to 2008, which suggested a figure of approximately 1,080,000 HTLV-1 carriers [9], similar to that reported previously. In addition, the number of ATL patients tends to increase as HTLV-1 carriers get older, and the numbers of patients in urban and suburban areas have increased [9]. Targeted health care-resource allocation is therefore urgently required to address the needs of ATL patients. The government introduced comprehensive HTLV-1 measures, including prevention, consultation, specialized institutions, research and development, in 2010 [10].

It is essential to estimate the number of treated patients and to quantify the efficacy and efficiency of patient care to improve the health care-delivery system. However, there have been few studies of the health care-delivery system in Japan. Regional differences in prevalence of ATL might also cause regional differences in physicians’ experiences and the efficiency of care-resource use. Although ATL is currently incurable, BMT should be available to suitable patients because as it enhances long-term survival [5]. However, there are regional differences in the use of BMT for ATL because of cost and the need for experienced physicians [11].

This study aimed to clarify the regional differences in the performance of BMT for ATL, and the differences in prognosis and care-resource use associated with different treatment patterns.

## Methods

### Study design, setting, and participants

This was a cross-sectional study using a Japanese hospital administrative database, the diagnostic-procedure combination/per diem payment system (DPC/PDPS). Japanese universal health care insurance is a system that provides every Japanese citizen with insurance benefits in cases of disease, injury, death, and childbirth. Every citizen must belong to one of the social insurance plans that are composed of three categories: Employee Health Insurance, District Health Insurance, and Elderly Health Insurance [12]. The health insurance funds gather premiums from their members and reimburse the costs of treatment. The reimburse system has long been based on a fee-for-service (FFS) using a national fee schedule. The enforcement of the same fee schedule for all insurance plans and almost all providers has maintained equity and contained costs, and the co-payment rate has become the same for all, except for elderly people and children [12].

However health care expenditure in Japan has steadily increased, and DPC/PDPS was introduced in 2002 to contain health expenditures [13] and to improve the quality of care in Japanese care facilities [14].

DPC/PDPS has been adopted across the plan, and is used mainly by research, educational and acute-care facilities throughout Japan. The DPC programme covers more than 90% of acute in-patient care in 2007 [13], and is increasing. The DPC/PDPS includes anonymous charge data, clinical data and care-process data, which can be analysed. This study represents the secondary use of DPC/PDPS collected from June 1st to December 31st 2010, conducted by MHLW.

Study subjects were patients admitted to a hospital for chemotherapy, whose primary diagnosis was classified as ‘C915’, corresponding to ATL in ICD10. Chemotherapy was defined as the use of chemotherapeutic agents used by ‘quality indicators programs’ in Japanese national hospitals. The demographical data were obtained from related bureaus. Data related to BMT was obtained from the Japan Society for Hematopoietic Stem Cell Transplantation [15]. The outline for this study followed the STROBE guidelines [16] and was approved by the ethics committee of the University of Occupational and Environmental Health in Kitakyushu, Fukuoka, Japan.

### Definition of variables

We used population, population density and proportions of aging of each region as demographic measures. We identified the number of hospitals, number of hospitals per inhabitants, number of beds, number of beds per inhabitants, number of DPC hospitals, number of DPC hospitals per inhabitants, number of beds in DPC hospitals, number of beds in DPC hospitals per inhabitants, number of academic hospitals, number of academic hospitals per inhabitants, total medical expenditures, and total medical expenditures per capita as a measure of care delivery systems in Japan. The number of BMT teams, BMT team densities [11], total number of allogeneic BMTs, BMT rate [11] and number of BMTs per team was used as a measure of care resources of BMT delivery in Japan.

**Figure 1:**
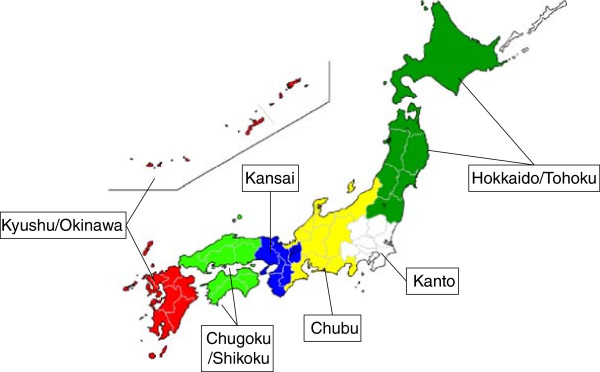
Definition of regions in Japan.

The variables used for analyses were gender, age, patient’s residence area, route of admission, hospital function, Charlson’s comorbidity index (CCI) [17], performance of BMT, in hospital mortality (IHM), length of stay (LOS) and total hospital charge (TC).Age was categorized as <65, and ≥65 years. Prefectures and regions (‘Hokkaido/Tohoku’, ‘Kanto’, ‘Chubu, Kansai’, ‘Chugoku/Shikoku’, and ‘Kyushu/Okinawa’) were defined according to the general rule in Japan (Figure 1). The route of admission was categorized as ‘elective’ or ‘urgent’. Hospital function was classified as ‘academic hospital’, including 80 university hospitals and two national centres, or ‘community hospital’. A CCI score of 0 indicated no comorbidities, and a score ≥1indicated the presence of comorbidities, excluding ATL as the primary neoplasm. The definition of BMT corresponded to ‘K922’ in the Japanese standardized fee-for-service payment system. We classified patients who received sequential chemotherapy and BMT in the BMT group.

We used IHM as index of outcome and TC and LOS as indices of care resource use. Although IHM was not regarded as an endpoint in general, we assumed that skilled experts could identify successful BMT compared with non-skilled individuals, thus in hospital mortality might be lower. TC (@JPY 100/USD) billed during admission was used as a proxy for cost [18]. In Japan, hospital charges are determined by the national uniform fee schedule. In this study, TC included physician fees, instrument costs, laboratory or imaging test costs, and administration fees.

### Statistical procedures

Data were analysed using χ^2^ tests for categorical variables and Kruskal-Wallis for continuous variables. Logistic regression analysis was used to determine the relationships between BMT and region, age, gender, route of admission, hospital function and comorbidity. The relationships between IHM and region, age, gender, BMT, route of admission, hospital function and comorbidity were determined by logistic regression analysis, and multiple linear regression analysis was used to quantify the effects of region, age, gender, BMT, IHM, route of admission, hospital function and comorbidity on LOS and TC. The distributions of both LOS and TC were right-skewed and these values were therefore log10-transformed in this model. The reference region for odds ratios (ORs) was Kyushu/Okinawa, because it had the highest prevalence rate of ATL. All statistical analyses were performed using PASW Statistics 18 (SPSS Inc., Chicago, IL, USA). All reported p values were two tailed, and the level of significance was set at 0.05.

## Results

The characteristics of each region are shown in Table 1. The density of inhabitants was concentrated in the Kanto and Kansai regions. Densities of hospitals, hospital beds, DPC hospitals and DPC hospital beds tended to be higher in the Chugoku/Shikoku and Kyushu/Okinawa regions than in the other regions examined. The densities of academic hospitals were higher in the Kanto region. The medical expenditures per capita were lower in the Kanto region. The team densities were lower in Kanto and higher in the Chugoku/Shikoku regions. The BMT rate was higher in the Kansai and Chugoku/Shikoku regions. The number of BMTs per team was highest in the Kanto and Kansai regions. The result of patient travelling to visit hospitals is shown in Table 2. Almost all patients were treated within their own region.

**Table 1:** Information of health care settings among each region

**Region**	**Population (million)**	**Population density (/km**^**2**^**)**	**The proportions of aging**	**Income per capita($)**	**N of hospitals**	**N of hospitals (per hundred thousand inhabitant**	**N of bed**
Hokkaido/Tohoku	14.8	100.7	25.3	23876.5	1236	8.3	226917
Kanto	42.6	1321.6	20.9	32110.8	2079	4.9	402613
Chubu	23.6	357.2	23.5	27739.8	1355	5.7	266617
Kansai	20.9	771.5	22.9	27413.1	1299	6.2	256673
Chugoku/Shikoku	11.5	228.0	26.2	25043.8	1187	10.3	194318
Kyushu/Okinawa	14.6	348.6	23.9	23288.4	1638	11.2	262265
**Region**	**N of bed (per thousand inhabitant)**	**N of DPC hospitals**	**N of DPC hospitals (per hundred thousand inhabitant)**	**N of bed of DPC hospital**	**N of bed of DPC hospital (per thousand inhabitant)**	**N of academic hospitals**	**N of academic hospitals (permillion inhabitant)**
Hokkaido/Tohoku	15.3	174	1.2	59320	4.0	13	0.9
Kanto	9.5	389	0.9	136169	3.2	76	1.8
Chubu	11.3	263	1.1	90415	3.8	25	1.1
Kansai	12.3	277	1.3	85334	4.1	20	1.0
Chugoku/Shikoku	16.8	163	1.4	48546	4.2	12	1.0
Kyushu/Okinawa	18.0	239	1.6	60437	4.1	17	1.2
**Region**	**Total of health care expenditure (billion dollar)**	**Total of health care expenditure per capita (dollar)**	**N of BMT team**	**Team densit(per 10million inhabitant)**	**Total AlloBMT**	**BMT rate (per 10million inhabitant)**	**N of BMT per team**
Hokkaido/Tohoku	35.4	2388.4	50	33.7	344	231.79	6.9
Kanto	83.2	1953.8	102	23.9	1055	247.63	10.3
Chubu	50.2	2131.9	78	33.1	458	194.31	5.9
Kansai	50.0	2390.4	78	37.3	625	299.00	8.0
Chugoku/Shikoku	30.9	2679.0	57	49.4	344	298.12	6.0
Kyushu/Okinawa	30.8	2728.9	60	41.1	398	272.66	6.6

DPC: Diagnosis Procedure Combination, LOS: Length of stay, BMT: Bone marrow transplantation.

**Table 2:** Matrix of patient travelling

**Hospital address**													
	**Hokkaido/Tohoku**		**Kanto**		**Chubu**		**Kansai**		**Chugoku/Shikoku**		**Kyusuhu/Okinawa**		**Total**
	**N**	**(%)**	**N**	**(%)**	**N**	**(%)**	**N**	**(%)**	**N**	**(%)**	**N**	**(%)**	
Patient residence													
Hokkaido/Tohoku	67	(100.0)	0	(0.0)	0	(0.0)	0	(0.0)	0	(0.0)	0	(0.0)	67
Kanto	0	(0.0)	130	(99.2)	0	(0.0)	0	(0.0)	0	(0.0)	2	(0.3)	132
Chubu	0	(0.0)	0	(0.0)	74	(98.7)	0	(0.0)	0	(0.0)	2	(0.3)	76
Kansai	0	(0.0)	0	(0.0)	1	(1.3)	174	(99.4)	2	(2.3)	0	(0.0)	177
Chugoku/Shikoku	0	(0.0)	0	(0.0)	0	(0.0)	0	(0.0)	84	(97.7)	0	(0.0)	84
Kyushu/Okinawa	0	(0.0)	1	(0.8)	0	(0.0)	1	(0.6)	0	(0.0)	652	(99.4)	654
Total	67	(100.0)	131	(100.0)	75	(100.0)	175	(100.0)	86	(100.0)	656	(100.0)	1190

The distribution of subjects by region is shown in Table 3. There were a total of 712 subjects, of whom 60.5% were Kyushu/Okinawa residents. There were significant relationships between region and gender, age, hospital functions and therapy. Subjects in the Kanto and Chubu regions tended to be younger than those in the Kyushu/Okinawa region. The proportions of academic hospitals tended to be higher in the Kanto and Chugoku/Shikoku regions compared with Kyushu/Okinawa. The proportions of patients treated with BMT tended to be higher in the Kanto and Kansai regions compared with the Kyushu/Okinawa region.

**Table 3:** Distribution and characteristics of subjects by region

**Region**														
		**Hokkaido/Tohoku (N = 32)**		**Kanto (N = 62)**		**Chubu (N = 45)**		**Kansai (N = 87)**		**Chugoku/Shikoku (N = 55)**		**Kyushu/Okinawa (N = 431)**		**p value**
Gender														
	Male (%)	17	(53.1)	41	(66.1)	26	(57.8)	33	(37.9)	29	(52.7)	219	(50.8)	0.028
	Female (%)	15	(46.9)	21	(33.9)	19	(42.2)	54	(62.1)	26	(47.3)	212	(49.2)	
Age														
	<65 (%)	13	(40.6)	40	(64.5)	24	(53.3)	48	(55.2)	28	(50.9)	184	(42.7)	0.012
	≧65 (%)	19	(59.4)	22	(35.5)	21	(46.7)	39	(44.8)	27	(49.1)	247	(57.3)	
Route of admission														
	Elective (%)	27	(84.4)	51	(82.3)	39	(86.7)	75	(86.2)	48	(87.3)	383	(88.9)	0.740
	Urgent (%)	5	(15.6)	11	(17.7)	6	(13.3)	12	(13.8)	7	(12.7)	48	(11.1)	
Hospital function														
	Community (%)	27	(84.4)	33	(53.2)	31	(68.9)	72	(82.8)	32	(58.2)	345	(80.0)	<0.001
	Academic (%)	5	(15.6)	29	(46.8)	14	(31.1)	15	(17.2)	23	(41.8)	86	(20.0)	
Comobidity														
	Absent (%)	18	(56.3)	36	(58.1)	25	(55.6)	50	(57.5)	35	(56.3)	260	(60.3)	0.950
	Present (%)	14	(43.8)	26	(41.9)	20	(44.4)	37	(42.5)	20	(43.8)	171	(39.7)	
Therapy														
	Chemotherapy (%)	30	(93.8)	51	(82.3)	40	(88.9)	66	(75.9)	53	(96.4)	408	(94.7)	<0.001
	BMT (%)	2	(6.3)	11	(17.7)	5	(11.1)	21	(24.1)	2	(3.6)	23	(5.3)	
Outcome														
	In hospital mortality (IQR)	8	(25.0)	8	(12.903)	7	(15.6)	15	(17.2)	13	(23.6)	91	(21.1)	0.519
	0.519 Length of stay (IQR)	40	(59.0)	50	(61.0)	39	(50.0)	48	(63.0)	35	(34.0)	32	(47.0)	<0.001^a^
	0.001a Total cost ($) (IQR)	1497.7	(1944.6)	2082.8	(5244.6)	1594.2	(3221.1)	1761.2	(5612.5)	1282.2	(2126.0)	1304.6	(1779.2)	<0.001^a^

^a^by Kruskal-Wallis test, other p values by chi-square test, BMT: Bone marrow transplantation.

The results of multivariate regression analyses are shown in Table 4. The variables significantly related to performance of BMT were living in the Kanto and Kansai regions (adjusted OR (AOR) 2.88 [1.20–6.89], AOR 6.30 [2.98–13.32], respectively, reference = Kyushu/Okinawa region), age (≥65; AOR 0.10 [0.04–0.24], reference = <65 years), academic hospital (AOR 1.93 [1.03–3.62], reference = community hospital) and comorbidity present (AOR 0.19 [0.09–0.43], reference = comorbidity absent).

**Table 4:** Results of multivariate regression analyses

		**BMT**			**IHM**			**LogLOS**			**LogTC**	
	**AOR**	**95% CI**	**p**	**AOR**	**95% CI**	**p**	**β**	**95% CI**	**p**	**β**	**95% CI**	**p**
Region												
Hokkaido/Tohoku	1.79	[0.36-8.92]	0.478	1.16	[0.50-2.73]	0.726	0.03	[-0.17-0.43]	0.400	0.02	[-0.19-0.14]	0.475
Kanto	2.88	[1.20-6.89]	0.018	0.49	[0.22-1.13]	0.094	0.11	[0.13-0.59]	0.002	0.10	[0.15-0.61]	0.001
Chubu	2.35	[0.77-7.15]	0.133	0.65	[0.27-1.55]	0.327	0.02	[-0.19-0.32]	0.606	0.01	[-0.23-0.28]	0.838
Kansai	6.30	[2.98-13.32]	<0.001	0.59	[0.31-1.13]	0.111	0.10	[0.08-0.47]	0.006	0.09	[0.09-0.48]	0.005
Chugoku/Shikoku	0.42	[0.09-1.89]	0.256	1.38	[0.69-2.77]	0.369	-0.01	[-0.26-0.21]	0.818	0.00	[-0.24-0.23]	0.961
Kyushu/Okinawa		reference			reference			reference			reference	
Gender												
Male		reference			reference			reference			reference	
Female	0.80	[0.44-1.46]	0.468	0.69	[0.47-1.01]	0.058	0.02	[-0.09-0.16]	0.606	0.03	[-0.07-0.18]	0.377
Ages												
<65		reference			reference			reference			reference	
≥65	0.10	[0.04-0.24]	<0.001	1.57	[1.03-2.38]	0.035	-0.02	[0.16-0.10]	0.637	-0.04	[-0.21-0.05]	0.245
Route of admission												
Elective		reference			reference			reference			reference	
Urgent	0.26	[0.06-1.17]	0.079	2.43	[1.46-4.04]	0.001	0.11	[0.10-0.48]	0.002	0.13	[0.23-0.60]	<0.001
Hospital function												
Community		reference			reference			reference			reference	
Academic	1.93	[1.03-3.62]	0.039	0.54	[0.32-0.91]	0.021	0.04	[-0.06-0.24]	0.246	0.12	[0.14-0.44]	<0.001
Comobidity												
Absent		reference			reference			reference			reference	
Present	0.19	[0.09-0.43]	<0.001	0.89	[0.60-1.33]	0.569	-0.02	[-0.16-0.09]	0.605	0.01	[-0.11-0.15]	0.755
Procedure												
BMT		-		4.61	[2.37-8.97]	<0.001	0.24	[0.55-1.02]	>0.001	0.45	[1.48-1.95]	<0.001
Outcomes												
IHM		-			-		0.27	[0.47-0.78]	<0.001	0.32	[0.71-1.02]	<0.001
Goodness of fit		0.738^a^			0.801^a^			0.187^b^			0.413^b^	

IHM: In hospitality, LOS: Length of stay, TC: Total charge, CTx: Chemotherapy, BMT: Bone marrow transplantation.

AOR: Adusted odds ratio, β: Standardized coefficient.

^a^:Hosmer-Lemeshow test, ^b^Coefficient of determination.

There were no relationships between performance of BMT and region or IHM. The variables related to IHM were age (≥65; AOR 1.57 [1.03–2.38], reference = <65) and urgent admission (AOR 2.43 [1.46–4.04], reference = elective admission), academic hospitals (AOR 0.54 [0.32–0.91], reference = community hospitals) and BMT (AOR 4.61 [2.37–8.97], reference = BMT not performed).

Related to logLOS, Kanto (standardized partial regression coefficient (β) 0.11 [0.13–0.59] and Kansai (β 0.10 [0.08–0.47]) regions had significantly longer LOS than that of the Kyushu/Okinawa region.

Other variables significantly related to logLOS were urgent admission (β 0.11 [0.10–0.48], reference = elective admission), BMT (β 0.24 [0.55–1.02], reference = BMT not performed) and IHM(β 0.27 [0.47–0.78], reference = no in hospital mortality).

Kanto (β 0.10 [0.15–0.61]) and Kansai (β 0.09 [0.09–0.48]) regions also had significantly higher logTC than that of the Kyushu/Okinawa region. Other variables related to logTC were urgent admission (β 0.13 [0.23–0.60], reference = elective admission), academic hospital (β 0.12 reference = community hospital), BMT (β 0.45 [1.48–1.95], reference = BMT not performed) and IHM (β 0.32 [0.71–1.02], reference = no in hospital mortality).

## Discussion

This study clarified the association between residence region and the proportion of patients with ATL who received BMT, IHM, LOS and TC. There were significant regional differences in performance of BMT as Kanto and Kansai regions had significantly higher rates of BMT performance. Although the Kyushu/Okinawa regions had a high prevalence of ATL, a lower proportion of patients were treated with BMT. Additionally, the Kanto and Kansai regions had high care resource use, while there was no significant difference in IHM between regions.

Previous studies in Western countries reported regional differences in the use of BMT were related to the prevalence of related diseases [11], per capita gross national income and per capita health care expenditures as economic factors, and the availability of BMT teams as care resources [19,20]. However, the current study found no significant relationship between the prevalence of ATL and the performance of BMT in Japan. Regarding economic factors, the health care expenditure per capita in the Kanto and Kansai regions, where higher numbers of BMTs were performed, was no higher than those of other regions. Reasons for the relatively lower health care expenditures observed could be age structure, that is, a lower proportion of elderly individuals in the Kanto and Kansai regions leading to lower health care expenditure, especially as there was no association between health care expenditure and performing BMT. However, there might be a relationship between household income and BMT, as demographic data showed that Kanto and Kansai regions were densely inhabited, and that their incomes seemed to be higher. There was no relationship between care resources, including the number of hospitals or BMT team density and performing BMT, which was in contrast to prior findings. However, the discrepancy between high numbers of BMTs performed and equivalent care resources in Kanto and Kansai regions might be attributed to the type of health care delivery system in Japan. The health care plan determined by local government in Japan was revised 5 years ago. The aim of the health care plan is to allocate health care resources equally in Japan. However, it focused on quantity rather than quality, in general. Thus, care resources in the Kanto and Kansai regions were almost equivalent to other regions. However, the number of BMTs performed per team was higher in the Kanto and Kansai regions. Thus, the efficiency of care might be superior in the Kanto and Kansai regions, although the quantity of care resources was equivalent between all regions.

Another finding was that higher care resource use did not lead to lower IHM. Although many studies have investigated relationships between care resource use and outcomes, there is still no definitive evidence. IHM used in this study were not relevant as outcome measures; outcome measures used in hemato-oncologic research are generally overall survival [21], or day 100 mortality, especially for BMT [22]. Another explanation might be there simply was no association between care resource and outcome. An additional potential reason might be diminishing health values as expenditure increases [23]. Further studies are required to determine definitive information.

An important finding of the current study was that the care system in Japan should enhance consolidation rather than increase quantity. Furthermore, the health care delivery system should be assessed by quality rather than quantity in Japan.

Finally, in Japan there has been no national epidemiologic research for approximately 10 years, despite the high prevalence of ATL. Therefore, it is important to monitor the prevalence of ATL to enable policy makers to plan preventive strategies. DPC/PDPS data or the national medical claims database could facilitate epidemiologic research, and we suggest that these databases should be used more widely.

There were some limitations to the methodology and interpretation of the current study. The data used were only gathered over a 7-month period in 2010, and therefore might not be fully representative. Another potential limitation is that DPC/PDPS data could not provide follow-up data for subjects, because the only available outcome was IHM. In addition, we were unable to discriminate between myeloablative and non-myeloablative BMT using the current data. Furthermore, we could not obtain data related to primary evaluation, prevalence of disease, transplant rate and proportion of patients with transplantation that would allow a comparison of ATL with other diseases with homogeneous distribution in Japan.

## Conclusions

There were significant regional differences in the performance of BMTs and resource use within Japan. ATL prevalence was not related to BMT performance, resource use or outcomes. Factors related to regional socioeconomics might affect the performance of BMTs and care resource use within Japan.

## Abbreviations

ATL: Adult T-cell leukaemia; HTLV-1: Human T-lymphotropic virus 1; MHLW: Ministry of health, Labour and Welfare; BMT: Bone marrow transplantation; DPC/PDPS: Diagnostic-procedure combination/per diem payment system; ICD10: International statistical classification of diseases and related health problems tenth revision; CCI: Charlson’s comorbidity index; IHM: In hospital mortality; LOS: Length of stay; TC: Total charge; OR: Odds ratio; AOR: Adjusted odds ratio.

## Competing interests

The authors declare no competing interests.

## Authors’ contributions

TM conducted literature search, reviewed articles, synthesized finding, and drafted the manuscript. AB and TN contributed the synthesis and provided substantive input and edit on draft of the manuscript.SM, KFs and KFj critically reviewed the manuscript. All authors read and approved the final manuscript.

## Pre-publication history

The pre-publication history for this paper can be accessed here:

http://www.biomedcentral.com/1472-6963/14/337/prepub
